# Factors Associated with Puppy Training Class Attendance

**DOI:** 10.3390/ani15172582

**Published:** 2025-09-02

**Authors:** Emma L. Buckland, Rachel H. Kinsman, Jessie Fitts, Rachel Casey, Séverine Tasker, Jane K. Murray

**Affiliations:** 1 Dogs Trust, London EC1V 7RQ, UK; 2 Ecology and Conservation Research Lab, University of the West of England, Bristol BS16 1QY, UK; 3 Bristol Veterinary School, University of Bristol, Bristol BA6 8DD, UK; 4 Mars Veterinary Health, Shirley B90 4BN, UK

**Keywords:** puppy, dog, socialisation, training, training class, puppy party, cohort, welfare, behaviour

## Abstract

Attending puppy training classes may help to improve the human–dog relationship and the overall behaviour and trainability of the dog, yet class attendance and the structure and content of classes are not well known. This study aimed to describe the size and structure of classes attended and reasons for non-attendance, and to identify factors associated with training class attendance, by owners of puppies under 19-weeks-old. In a sample of 2187 owners participating in the ‘Generation Pup’ study, 67% reported attending at least one training class. Factors associated with increased odds of class attendance were higher household income, previous intention to attend, first-time ownership, and/or those who received a puppy information pack at acquisition. The likelihood of attending decreased as the acquisition age of the puppy increased. Classes were reported to vary in relation to the number, age, and size of puppies. Class content also varied, for example, in relation to opportunities for puppies to play with each other and training advice given. Common reasons for non-attendance included owners wanting to work with the puppy themselves and/or no suitable classes being available. These data may help to better understand training class attendance for young dogs and could inform strategies to encourage attendance amongst the dog-owning population.

## Simple Summary

Without careful exposure to positive early-life experiences, dogs may develop undesirable behaviours which may negatively impact their welfare and/or the bond with their owner. In-person training classes for puppies can provide some of these experiences as well as guidance on dog training techniques. However, little is known about class attendance, their structure or content. Such knowledge would help in considering how to boost attendance for classes and understand their impact. In a sample of 2187 dog owners participating in the ‘Generation Pup’ study, 67% of owners attended at least one class before their puppy reached 19-weeks-old. Owners were more likely to attend classes if they had a higher household income, were first-time dog owners and/or had received a puppy information pack. Owners were more likely to attend if they had previously reported an intention to attend. Conversely, attendance was less likely as the age of their puppy when they acquired it increased. Class structure and content were reported to vary, suggesting that the experiences provided by classes differed. Common reasons for non-attendance included owners wanting to work with the puppy themselves and lack of suitable classes.

## 1. Introduction

Early life training and careful exposure to new experiences are critical to a puppy’s behavioural development [1,2,3,4,5]. This is especially important for the human–dog relationship, since management and training of dog behaviour is key to successful ownership. Unwanted or undesirable behaviours are often cited as reasons for relinquishment [6,7,8,9] or euthanasia of otherwise healthy pet dogs [10,11]. Undesirable behaviours can also negatively affect the welfare of the dog [12,13,14] and the wellbeing of the owner [15]. Attendance at training classes has been demonstrated to improve the human–dog relationship [16] and improve the overall behaviour of the dog (e.g., obedience; [17,18]), which may in turn reduce the development of undesirable behaviours.

Behavioural learning and adaptive flexibility occur early in a puppy’s development—starting with neonatal and transitional periods and continuing to the sensitive period of socialisation which occurs from 3 to 12 weeks of age (depending on breed and individual [1]). Here, synaptic plasticity allows for rapid learning during new experiences, and it is during this period that puppies learn to associate social and non-social stimuli with positive or negative emotions and build adaptive capacity for the future [1]. Advice follows that the puppies should be gradually exposed to new stimuli and environments that they may encounter as household pets, including meeting new people and dogs, and handling [1,19]. Although socialisation and learning continue throughout a dog’s life, the first 6 months of a dog’s life are likely to be the most important for the prevention of unwanted behaviours in juvenile and adult dogs [20,21].

Puppy classes are designed for puppies aged 12–16 weeks. Classes typically aim to positively introduce puppies to the kinds of stimuli and social experiences that they are likely to encounter as household pets and to encourage development of appropriate behaviours that are learnt and reinforced [22]. These classes also provide valuable opportunities to provide support to owners of young puppies on the best practices for health and welfare. Training classes can be implemented as a single class or a series of classes [23]. The terms “puppy classes” and “puppy parties” are used interchangeably in practice [24], although the latter, anecdotally, may be less focused on training and behaviour skills and more commonly used as an opportunity to allow puppies to interact with each other. For this paper, the term “puppy class” will be used to describe both.

Seksel et al. [25] reported limited effects of training and/or socialisation, with only higher rates of responsiveness to commands in puppies in both the socialisation plus training and training groups compared to control groups. However, several studies have reported associations between participation in puppy or juvenile training classes and more favourable scores in sociability, trainability and handling later in life [26,27]. Non-attendance at training classes could be considered as a potential risk factor in the development of behavioural issues. For example, Lord et al. found that owners who did not attend puppy classes were more likely to report problem behaviour in their dogs at 9 months of age [28].

Since the number and proportion of dog owners attending puppy classes within the United Kingdom (UK) or the Republic of Ireland (ROI) are unknown, it would be useful to quantify attendance and what factors influence class attendance. In a small sample of 145 dog owners across Canada and the United States, Cutler et al. reported 49% of owners attended at least one puppy training class by age 20 weeks [29]. Training class attendance is encouraged by behaviour and veterinary professionals alike [30], yet the delivery of classes is not regulated, and they vary in size, content, and structure [29], with no studies (to our knowledge) describing the characteristics of such classes in the UK. Such variation in class content and structure may contribute to differences in the positive and potentially negative experiences of these classes, which is an important confounder to studies seeking evidence on their long-term impact.

The aims of this paper were to examine training class attendance in a cohort of puppy owners in the UK and ROI, identify dog and owner characteristics associated with attendance, describe the size, structure and variation within classes attended, and explore the reasons for non-attendance. The objective of this study was to better understand training class attendance and to inform strategies to encourage attendance among puppy owners.

## 2. Materials and Methods

### 2.1. Study Participants and Design

‘Generation Pup’ (https://generationpup.ac.uk/—accessed on 5 August 2025) is a longitudinal study which follows dogs of any breed or mix of breeds. To join, participants must be: (1) a resident in the UK or ROI, (2) at least 16-years-old and (3) own a puppy under 16-weeks-old at time of registration (or under 21 weeks if the puppy had been through import quarantine).

Upon registration to ‘Generation Pup,’ participating dog owners complete three mandatory surveys (age of the puppy at registration varied from birth to 16 weeks). All self-administered surveys are completed online or via postal surveys depending on owner preference. Following the initial mandatory surveys, owners are asked to complete a series of optional surveys at regular intervals throughout the lifetime of their enrolled dog [31]. The surveys include open and closed questions to examine various areas of health and behaviour that may affect the welfare of dogs. The protocol for the ‘Generation Pup’ study and survey timeline is described in detail in Murray et al. [31] (see Supplementary Materials).

### 2.2. Data Collection and Study Size

For this study, data were gathered from the three mandatory Registration surveys, and from three optional surveys which included specific questions regarding puppy training class attendance—here referred to as the ‘Settling In,’ ‘12-week’ and ‘16-week’ surveys. The ‘Settling In’ survey was issued to owners 1–3 weeks after the puppy joined the household and is available for 14 days or until 12 weeks of age, whichever was sooner. The ‘12-week’ and ‘16-week’ surveys were issued when the puppy reached each age point and were available to complete within 24 days.

Responses were combined across surveys, and the outcome variable was calculated as attendance (yes/no) of at least one class at or before 19 weeks of age (as the age of the puppy when the ‘16-week’ survey could be completed ranged from 16 to 19 weeks 3 days). Thus, this equates to each puppy having one set of survey responses. For owners with multiple puppies enrolled in ‘Generation Pup,’ data for one puppy was randomly selected for inclusion in the sample to avoid clustering bias. As UK COVID-19 lockdown restrictions were announced on the 23 March 2020 [32], and this may have impacted an owner’s ability to attend a puppy class (due to the cancellation of in-person training classes during lockdown), data collected after the commencement of lockdown were excluded from the analysis. Data were therefore gathered from completed surveys between 16 May 2016 and 22 March 2020.

### 2.3. Data Analysis

Attendance at one or more training classes was recorded for puppies by the age of 19 weeks (i.e., as reported across the three optional surveys). Univariable and multivariable logistic regression models were used to identify factors associated with attendance, using a binary outcome variable; puppies who attended at least one puppy class and puppies who did not attend a class by 19 weeks of age.

Characteristics of attending owners (age, gender, urban/rural location, housing tenure, annual household income, employment status, working in a dog-related industry, previous dog ownership, number of household adults, children and dogs) and puppies (acquisition age, sex, acquisition source, breed status and breed group [33]) were summarised into categorical or continuous explanatory variables (Table 1). In addition, owners’ responses regarding their intention to take their dog to puppy classes and whether they received a puppy pack when acquiring their puppy were included in the analysis (from Registration surveys). Within variables, where categories had few data points, univariable analysis was used to merge logically related categories with comparable associations to the outcome. For all responses, where owners responded “I prefer not to say” or left blank responses, these data were classified as missing. Free-text responses (e.g., in “other, please specify”) were coded into categorical variables by authors (E.L.B, J.F.) and spot checked (15%) for agreement. Codes were discussed and reassigned until agreement was reached, and a final check of the full data was completed to ensure agreement.

Univariable logistic regression was used to identify which of the above explanatory variables were associated with puppy class attendance (*p* < 0.2). These were then included in the multivariable logistic regression model building process which used backwards stepwise elimination. A complete case analysis was used, with missing data excluded by casewise deletion. The final model significance was set at *p* < 0.05, and the Hosmer-Lemeshow test was used to evaluate the model fit quality. The final model was assessed for confounding variables and collinearity of variables. Confounding variables were determined by building the model in a stepwise fashion and looking for changes in coefficients that were >20% [34]. Collinearity was judged by evaluating correlation matrices, variance inflation factors and tolerance [35]. The statistical packages IBM SPSS Statistics v26 (IBM Corp, Armonk, NY, USA) and R software version 4.1.3 (R Core Team, Vienne, Austria) were used for data analyses.

For those owners attending puppy classes, characteristics of the last class attended were summarised descriptively (vet practice or other provider, number of dogs in class, age and size of other dogs, type of training delivered at class [recall, sit, house training, introducing people], type of experience at class [firework sounds, interaction with adults or children] and the advice given at class [worming, vaccination, weight, neutering, health care, training, behaviour problems]). Finally, where owners did not attend a class, reasons for not attending were reported. Here, owners could select multiple responses, and a free text “other, please specify” option was also available.

## 3. Results

At the time of this study, recruitment to ‘Generation Pup’ was still ongoing, thus the analysis presented here used data gathered from 2187 puppies and their owners following data cleaning. Owner and dog characteristics are described in Table 1. Most responding owners were female (90.4%), employed (69.8%), did not work with dogs (86.6%) and had owned a dog before or currently owned another dog (70.2%). The age of respondents was distributed roughly evenly across four categories with a slight majority in the 16–34-year category (29.2%). The median (25th–75th percentile) number of adults in the household was 2 (2–2), children 0 (0–0) and other dogs 0 (0–1). The annual household income was ≥£55,000 for 36.5% of owners, and 64.7% of owners lived in a suburban, village or small-town area. The sex of the dog was evenly split (male: 50.8%) and the majority were of a specific breed (67.5%). Of those breeds, 28.9% were grouped into the Gundog category of Kennel Club groupings. Most dogs were acquired when ≥8 weeks of age (74.9%). At the time of acquisition, 58.2% of owners were provided with a puppy pack and most owners intended to attend classes with their enrolled puppy (83.0%).

Over two-thirds of owners reported that their puppy had attended at least one training class (67.1%; 1468) by the ‘16-week survey’ timepoint. Of the 719 owners who had not attended a puppy class, 37.6% (270) reported they intended to at some point.

Of the 17 explanatory variables assessed for association with puppy class attendance during univariable logistic regression, 10 with a *p*-value of <0.2 were included in the multivariable logistic regression model building process. These variables were Owner gender (Female/Male); Home location (City or urban area/Rural or suburban/Remote area); Previous dog ownership (Has a dog before or currently have another dogy/Never had a dog as an adult); Number of dogs in household (continuous variable); Annual income (continuous variable); Intention to attend puppy class (Not intended/Intended to go to a class); Puppy pack given to owner (Given a pack/Not given a pack); Acquisition age (continuous); Dog sex (Female/Male); Dog breed (Working dogs, all other group types and Non-recognised breed/mixed breeds). The final multivariable model identified five variables (*p* < 0.05) associated with puppy class attendance (Table 2). These were annual household income, the age of the puppy when acquired, receipt of a puppy pack when the puppy was acquired, the owner’s intentions regarding attending puppy classes, and dog ownership experience. The Hosmer-Lemeshow test indicated an acceptable model fit (*p* = 0.503) and no confounding variables or collinearity were detected in the model.

**Figure 1:**
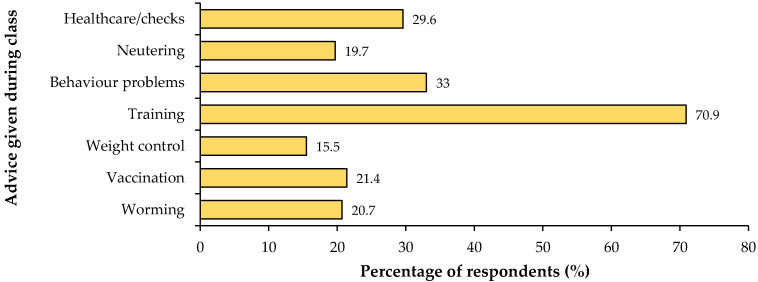
Frequency of owners who were provided with advice on topics (worming, vaccination, weight control, training, behaviour problems, neutering and health care/checks) during the class (*n* = 1468).

**Figure 2:**
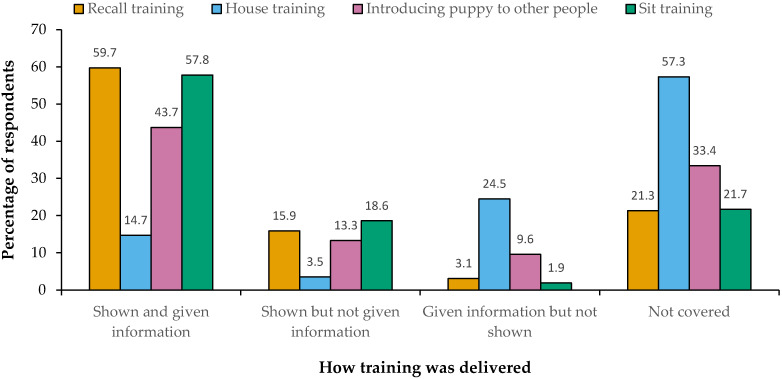
Frequency of how training (recall, sit, house training, introducing to other people) was delivered in the last class attended (*n* = 1468).

Owners who had attended one or more puppy classes (*n* = 1468) described the characteristics and learning experiences of their last class attended (Table 3), including the content covered (Figure 1) and the delivery of information about how to introduce their puppy to other people, recall training, house training and sit training (Figure 2). Of the 719 owners who had not reported attending a puppy class, 519 (72.2%) owners selected one or more reasons for non-attendance from a pre-defined list. The majority (57.4%; 298) of owners reported they wanted to work with the puppy themselves and a further 40.9% (212) reported there were “no suitable classes”. Other reasons for not attending were that they did not have time (11.6%; 60), they did not think the classes would help (9.6%; 50) and/or classes would not suit their puppy (5.8%; 30).

## 4. Discussion

This study summarised the characteristics of puppy classes attended and investigated dog and owner characteristics associated with class attendance. Up to the age of 19 weeks (at the ‘16-week’ survey), over two-thirds of owners reported attending at least one training class with their puppy, and a third of non-attending owners reported an intention to attend classes in the future. Longitudinal studies can be subject to selection bias, where factors such as socio-economic status, education level, and gender can influence participation [36]. Puppy class attendance within the ‘Generation Pup’ cohort was higher than has been reported in the previous literature. For example, Blackwell et al. described that most dog owners (88% of 192 surveyed) undertook training, but that the majority (58%) of those owners had used informal methods (‘trained their dog at home’) rather than formal classes [37]. In a Canadian study, only half (49% of 296) of owners surveyed took their puppy to structured classes [29]. Compared to these cross-sectional studies, it is plausible that since longitudinal studies—such as ‘Generation Pup’—require substantial commitment from participants, they may attract owners who are more inclined to attend classes. Indeed, ‘Generation Pup’ participants are recruited to the study via multiple methods, including via dog trainers and veterinary practices [31], which could have led to an over-representation of puppy class attendance in our cohort. The potential influence of these factors on puppy class attendance must be considered, and although the study benefits from a greater sample size, caution is needed before the findings are extrapolated to the wider dog-owning population.

Common reasons for non-attendance included wanting to work with the puppy themselves and no suitable classes. Although general training (i.e., at home by the owner themselves) may be useful, previous research has shown that formal or structured training may be of most benefit in improving obedience and behaviour [17,25]. A lack of suitable classes requires further exploration, as this was the wording of the response option provided to owners and the meaning of ‘suitable’ is open to interpretation. It could infer high demand of classes in areas (being fully booked), lack of classes of a perceived quality, gaps in the nationwide provision of classes or it may indicate a need for more accessible classes (e.g., regarding travel time to classes, time of day when classes are held, ages of puppies allowed to attend).

Several factors were associated with increased odds of attending classes, including household income, intention to attend and those who were first time dog owners. The effect of income in our study agrees with the findings of Cutler et al., who reported dog owners residing in lower income areas were less likely to attend classes than those living in higher income areas [29]. Since classes usually involve a fee, plus associated costs to attend in person, one explanation is that those with a higher household income are more likely to have the financial means to afford class attendance. However, while household income is often used as a proxy indicator for deprivation [38], there may be other socio-economic factors that would be valuable to explore in relation to dog training class attendance. For example, Harris et al. found high dropout rates for free online and in-person dog training classes in economically deprived areas, suggesting that barriers, other than or in addition to cost, exist [39].

New puppy ownership can be a challenging and busy time [40]. Our findings suggest that those who reported intentions of attending classes (at or before acquisition of their puppy) had increased odds of class attendance. Furthermore, 38% of owners who did not attend also reported that they had planned to attend in the future. These data may demonstrate intentional pre-planning and a commitment for class attendance in the early stages of puppy acquisition. However, it remains unclear when owners formed this intention or how long they held it before attending. For example, some owners may have already been enrolled in or attended classes when they expressed their intentions.

First time dog owners (those who had not owned a dog as an adult before acquiring their puppy) had increased odds of class attendance compared to those who had previously or currently owned another dog. This may suggest that classes better appeal to first time owners than those that have owned dogs before. Indeed, the benefits of class attendance on the dog per se versus the owner (for advice and training support) warrant further investigation. First time dog owners have been reported to have less understanding of dog behaviour [41], and to report more undesirable behaviours in their dog(s) [42], thus attendance at classes is likely to have a greater positive impact for these new owners.

Owners who received a puppy pack when acquiring their puppy had increased odds of class attendance compared to those who did not receive a pack. Though this study did not seek to define what a puppy pack is or specifically what participants received, it is widely accepted that a pack may include leaflets or guidance and/or items for looking after a puppy and advice on training, as supplied by breeders or rehoming organisations [43]. Therefore, these packs, though they likely vary in content, may be successful in increasing class attendance. However, it may also be explained that those who acquire puppies from reputable sources (which are more likely to provide packs), may also seek out recommended ownership practices such as training classes.

As the age of puppies at acquisition increased, the likelihood of class attendance decreased. This may be due to a few practical factors, for example, classes typically start at 12–16 weeks of age and for dogs acquired at this age, there may be less availability to book onto classes at short notice or owners may have wanted to have a settling in period before attending a class setting. In addition, dogs acquired later may be less likely to have completed their vaccination course (often a prerequisite for class attendance) [23] since the second dose can be received up to 16 weeks of age [44]. No further dog characteristics (UK Kennel Club group, breed status or sex) influenced the likelihood of class attendance. However, it is reported in the previous literature that breeds of dogs are perceived to differ in trainability [45] and that larger dogs may be more likely to have received obedience training [42], thus investigation of class attendance in relation to breed and size of dog is recommended for future studies.

In this study, owners reported the characteristics of the last class they had attended, as a representation of the type of class they attended (since many classes are run as a set) (Table 3). Our findings show that classes varied widely in size and structure and in the type of information and opportunities provided. Dog training is an unregulated profession and there are no set guidelines on class provision [30] and this variation in class structure is documented in other literature [46]. To minimise potential recall bias, information on class content and structure was only collected for the class that was most recently attended, and this limits the interpretation of the findings as representative of all classes attended. Indeed, this question design may have skewed our findings to the last class attended and there may be a tendency to deliver certain content during different stages within a series of classes. Our study did not ask for more detail on the number of classes attended or the content covered over multiple classes, so this detail is unknown. The main content of classes attended in our study included basic training skills, since recall and sit techniques were provided in over half of classes, yet house training advice was not provided for 57% of participants (Figure 1), suggesting basic training is not always comprehensively covered. Most classes were run outside of veterinary practices, and this may be a missed opportunity to set up positive experiences within the veterinary setting. Fear of veterinary procedures is an important welfare issue which inhibits the ability to provide effective preventative treatment and medical care [47], and early positive associations are a key factor in mitigating fear within a veterinary setting [48]. The lack of provision of classes by veterinary practices may also provide a potential explanation for the relatively low proportion of health content reported in classes (for example, discussions of weight control, parasitic control, vaccination, and neutering). Indeed, Christo and Buckley reported that although veterinary-led puppy classes provided information on behaviour topics, the staff undertaking them lacked training and behaviour qualifications [46]. These findings highlight the potential for inconsistent content or missed opportunities across different providers. In this study, data were not gathered on the type of non-veterinary providers or the qualifications/accreditations of training providers or the cost of attending classes and this may be useful information for future work.

The emotional valence induced by the socialisation or habituation experience provided is as important as the provision of such experiences, since the emphasis of the benefit of such classes is on their positive nature [30]. The structure and content of classes, including opportunities for interaction with unfamiliar dogs or people, is therefore important to enable positive, and avoid negative, experiences. In this study, the owners’ perception of the experience during classes was not determined; however, there was considerable variation in the structure and opportunities for interactions, which may infer variation in positive experiences within classes. For example, almost all classes had between 1 and 10 dogs, but it is notable that 6.5% had 11 or more dogs which is a large number of dogs in one setting, especially in managing positive experiences for all dogs. For over half of the classes, dogs were the same age and mixed in size. Opportunities for dog-dog interaction were mixed; in 42% of classes, puppies did not play together and in 25% of classes some individual puppies were allowed to play together. This contrasts with interactions with people, where most classes included interaction with one or more adults (which may include the class leader). Though this study did not intend to evaluate the in-depth experiences within attended classes, variation in these experiences is an important, yet overlooked, factor that can influence the impact of class attendance on future behaviour or training [48,49]. It is plausible that any such variability may contribute to the mixed evidence of the benefits of classes on training and behaviour in later life, as found within the literature. The specific experiences of dogs during classes on future dog behaviour, trainability, and the human–animal bond should be explored further. In the long term, the project within which these data were generated, ‘Generation Pup,’ offers the ability to investigate class attendance on the long-term welfare, behaviour, and health of dogs.

## 5. Conclusions

Our study found that though most owners attended at least one training class with their puppy by 19 weeks of age, there were several owner-related factors associated with increased odds of attendance, and the structure and content of classes varied widely. It is useful to understand the characteristics of those attending vs. non-attending owners and of the content of classes. Further studies could evaluate the impact of the class experience on long-term training and behaviour, as well as explore new initiatives to encourage class attendance among those who did not attend. These initiatives could include combining tailored educational or media campaigns with behavioural change techniques, such as using trusted stakeholders (e.g., welfare organisations, veterinary bodies, and/or public figures) within the community to help shift social norms so that attending puppy classes becomes an increasingly common practice.

## Figures and Tables

**Table 1:** Descriptive statistics of variables assessed as factors potentially associated with puppy class attendance by owners of puppies under 19-weeks-old (*n* = 2187). (No missing data unless stated).

**Owner Characteristics**			**Dog Characteristics**			
**Variable**	**Category**	***n*** **(%)**	**Variable**	**Category**	***n*** **(%)**	
Owner gender	Female	1970 (90.4)	Sex of dog	Female	1076 (49.2)	
(missing data = 7)	Male	210 (9.6)		Male	1111 (50.8)	
Owner age (missing data = 6)	16 to 34 years	637 (29.2)	Acquisition age	<56 days	549 (25.1)	
	35 to 44 years	449 (20.6)		≥56 days	1638 (74.9)	
	45 to 54 years	513 (23.5)	Source of puppy (missing data = 201)	Kennel Club assured/professional breeder	864 (43.5)	
	55 and more years	582 (26.7)				
Employment status (missing data = 22)	Not working Employed	653 (30.2) 1512 (69.8)				
				Hobby/occasional breeder	996 (50.2)	
Owner works with dogs (missing data = 675)	No Yes	1309 (86.6) 203 (13.4)		Home bred	11 (0.6)	
				Rehomed	115 (5.8)	
			Breed status	Specific named breed	1476 (67.5)	
Previous dog ownership experience (missing data = 1)	Had dog before/	1534 (70.2)		Cross of two breeds	525 (24.0)	
	currently have			(e.g., Labradoodle,		
	another dog			Collie X Labrador,		
	Never had a dog as	652 (29.8)		Cavachon)		
	an adult			Mixed breed/unknown	186 (8.5)	
Number of household adults (missing data = 114)	1	264 (12.7)		breed or mixed breed		
	2	1330 (64.2)		without known		
	3	285 (13.7)		parentage, but of a		
	4	151 (7.3)		particular ‘type’ (e.g.,		
	5 or more	43 (2.1)		Collie type/collie cross)		
Number of household children (missing data = 114)	0	1624 (78.3)	UK Kennel Club Group	Gundog	631 (28.9)	
	1	217 (10.5)		Hound	99 (4.5)	
	2	180 (8.7)		Pastoral	238 (10.9)	
	3 or more	52 (2.5)		Terrier	164 (7.5)	
Number of other household dogs	0	1291 (59.0)		Toy	83 (3.8)	
	1	511 (23.4)		Utility	141 (6.4)	
	2	222 (10.2)		Working	70 (3.2)	
	3 or more	163 (7.5)		Non-recognised	761 (34.8)	
Annual household Income (missing data = 366)	Less than £15,000	116 (6.4)		breed/mix breed		
	£15,000–<£25,000	232 (12.7)	**Other variables**			
	£25,000–<£35,000	263 (14.4)	**Variable**	**Category**	***n*** **(%)**	
	£35,000–<£45,000	301 (16.5)	Puppy pack given	Given a pack	1273 (58.2)	
	£45,000–<£55,000	274 (15.0)	during acquisition	Not given a pack	914 (41.8)	
	£55,000 or more	664 (36.5)	Intention to attend	Yes, intended to attend	1816 (83.0)	
Urban/rural location (as described by owner) (missing data = 6)	Remote area	58 (2.7)	puppy class	a class		
	Rural area	360 (16.5)		No intention to	371 (17.0)	
	Suburban, village/	1412 (64.7)		attend a class		
	small town					
	City/urban area	351 (16.1)				

**Table 2:** Multivariable logistic regression model of odds ratios (ORs), 95 per cent confidence intervals (CIs) and *p* values of explanatory variables significantly associated with puppy class attendance by owners of puppies under 19-weeks-old.

Variable	Category	One or More Classes Attended *n* (%)	Not Attended *n* (%)	OR (95% CI)	*p* Value
Annual household income	Continuous ^(a)^			1.11 (1.04–1.19)	0.002
Acquisition age in days	Continuous			0.99 (0.98–0.99)	<0.001
Intention to attend puppy class	Not intended	77 (20.8)	294 (79.2)	1.00	
	Intended to attend a class	1391 (76.6)	425 (23.4)	11.80 (8.66–16.08)	<0.001
Owner received puppy pack	Given a pack	919 (72.2)	354 (27.8)	1.00	
	Not given a pack	549 (60.1)	365 (39.9)	0.61 (0.49–0.77)	<0.001
Dog ownership experience	Had a dog before or currently have another dog	985 (64.2)	549 (35.8)	1.00	
	Never had a dog as an adult	482 (73.9)	170 (26.1)	1.33 (1.04–1.70)	0.021

^(a)^ Owners could select one of the following income brackets: “<£15,000”, “£15,000–<£25,000”, “£25,000–<£35,000”, “£35,000–<£45,000”, “£45,000–<£55,000”, “£55,000 or more”. For the analysis, this variable was treated as a continuous variable by using the middle value of the brackets and an upper income of £75,000.

**Table 3:** Descriptive statistics of the last puppy class that owners who attended one or more classes went to (*n* = 1468). (No missing data unless otherwise stated).

Variable	Category	*n* (%)
Number of dogs in class (missing data = 2)	1–5	768 (52.4)
	6–10	601 (41.0)
	11–15	77 (5.3)
	16–20	17 (1.2)
	20+	3 (0.2)
Age of dogs in class (missing data = 6)	The same age as puppy	789 (54.0)
	Older than puppy	174 (11.9)
	Younger than puppy	38 (2.6)
	Mixed ages	461 (31.5)
Size of other dogs in class	Mainly smaller than puppy	260 (17.7)
	Mainly larger than puppy	276 (18.8)
	Mixed sizes	932 (63.5)
Class run by a veterinary practice	Yes	286 (19.5)
	No	1182 (80.5)
Interactions with other dogs	The puppies all played together	492 (33.5)
	Some individual puppies were put together to play	364 (24.8)
	Puppies did not play together	612 (41.7)
Interactions with people	Interacted with one or more adults	1381 (94.1)
	Interacted with one or more children	504 (34.3)
Socialisation recommendations given	Give puppy experience of as many new things as possible before 12 weeks of age	829 (56.5)
	Introduce puppy to new things gradually	434 (29.6)
	Not covered	205 (14.0)
Sound of fireworks	Heard the sounds of noises such as fireworks	151 (10.3)
	Not covered	1317 (89.7)

## Data Availability

The data are not publicly available due to ethical approval of participant informed consent that included “Generation Pup” participants being informed that we will remove all personally identifiable information before sharing data with universities and/or research institutions.

## Supplementary Materials

The study method and surveys can be found at Murray et al. [31] (https://doi.org/10.1186/s12917-020-02730-8).
